# Prices of over-the-counter drugs used by 15-year-old adolescents in Germany and their association with socioeconomic background

**DOI:** 10.1186/s12889-017-4923-2

**Published:** 2017-11-25

**Authors:** Salvatore Italia, Silke B. Wolfenstetter, Irene Brüske, Joachim Heinrich, Dietrich Berdel, Andrea von Berg, Irina Lehmann, Marie Standl, Christina M. Teuner

**Affiliations:** 1 Department of International Health, School CAPHRI, Care and Public Health Research Institute, Maastricht University, PO Box 616, 6200 MD Maastricht, The Netherlands; 2 Helmholtz Zentrum München, German Research Center for Environmental Health, Institute of Health Economics and Health Care Management, Ingolstädter Landstraße 1, 85764 Neuherberg, Germany; 3 Helmholtz Zentrum München, German Research Center for Environmental Health, Institute of Epidemiology I, Ingolstädter Landstraße 1, 85764 Neuherberg, Germany; 4 Marien-Hospital Wesel, Research Institute – Department of Pediatrics, Pastor-Janßen-Straße 8-38, 46483 Wesel, Germany; 5 UFZ – Helmholtz Centre for Environmental Research Leipzig, Department of Environmental Immunology, Permoserstr. 15, 04318 Leipzig, Germany

**Keywords:** Adolescent, Drug utilization, Drug prices, Socioeconomic factors, Over-the-counter drugs

## Abstract

**Background:**

In Germany, over-the-counter (OTC) drugs are normally reimbursed up to the age of 12 years only. The aim of this study was to analyse prices of over-the-counter drugs used by adolescents in Germany and their association with socioeconomic factors.

**Methods:**

Based on the German GINIplus and LISAplus birth cohorts, data on drug utilization among 15-year-old adolescents (*n* = 4677) were collected using a self-administered questionnaire. The reported drugs were subdivided into prescription drugs and OTC drugs. The drugs’ prices were tracked by the pharmaceutical identification numbers.

**Results:**

Overall, 1499 OTC drugs with clearly identifiable prices were eligible for analysis. Their mean price was €9.75 (95% confidence interval: €9.27–10.22). About 75% of the OTC drugs cost less than €10. Higher mean prices were associated with residing in Munich (€10.74; 95% confidence interval: €9.97–11.52) and with higher paternal education (e.g. highest education level: €10.17; 95% confidence interval: €9.47–10.86). Adolescents residing in Munich (in comparison with the less wealthy region of Wesel) and adolescents with higher educated fathers were also significantly more likely to use OTC drugs costing ≥ €10 or ≥ €25, respectively.

**Conclusions:**

The price of €10 for non-reimbursable OTC drugs may represent a (psychological) threshold. Higher prices could discourage especially adolescents from a lower socioeconomic background from taking medically advisable but non-reimbursable OTC drugs.

## Background

In Germany, drugs available without a physician’s prescription are not normally covered by statutory health insurance for children aged ≥12 years. This regulation affects pharmaceuticals with defined chemical active ingredients such as ibuprofen or acetylcysteine as well as herbal drugs or homeopathic preparations, regardless of whether the medicinal products can provide evidence for efficacy or not. As in Germany, over-the-counter (OTC) drugs are not reimbursed in many other European countries as well [1]. Many OTC drugs are useful, but socioeconomic barriers might finally result in adolescents (and adults) not taking medically advisable (especially higher priced) OTC drugs in case of illness or disorder if the drugs have to be paid for out of pocket. Furthermore, according to various studies, OTC drugs can provide value to society if taken in time, for example by avoiding costs for physician visits and cost-intensive prescription drugs or maintaining the population’s working productivity [2–4]. However, the prevalence of OTC drug use among adolescents has been found to be significantly associated with socioeconomic factors such as parental education or place of residence [5, 6]. To our knowledge, there are no data on how socioeconomic factors are correlated with the prices of OTC drugs used by adolescents in Germany or other countries. Therefore, the main objective of this study was to analyse whether there was an association between drug prices and socioeconomic factors with regard to OTC drugs used by 15-year-old adolescents from the German GINIplus and LISAplus birth cohorts.

## Methods

### Study population

The GINIplus and LISAplus studies are two German birth cohorts that started with 5991 (GINIplus) and 3097 (LISAplus) healthy full-term newborns who were recruited between September 1995 and January 1999 from obstetric clinics in the southern (Munich), eastern (Leipzig), and north-western (Bad Honnef and Wesel) parts of Germany [7]. In both studies, only children with a birth weight of ≥2500 g were included. Participants with insufficient German language skills were not eligible for both studies. Furthermore, children with non-German parents or parents born outside Germany were excluded from the LISAplus study.

### Data collection

For the 15-year follow-up, exactly 6094 participants’ parents/legal guardians were contacted between January 2011 and October 2014. Using a self-administered questionnaire, information on drug utilization was collected retrospectively for four weeks prior to questionnaire completion. The procedure used in several big German surveys (e.g. KORA [8], KIGGS [9]) is to ask recall of drug use one week prior to questionnaire completion to reduce recall error. We extended this period to reduce misclassification. A longer timeframe seemed, however, not suitable to us, as adequate recall of drugs used not regularly would be too difficult for parents, especially if they have more than one child. However, as data collection stretched over several seasons, seasonal effects are not to be expected. Participants were asked to enter the drug names and the pharmaceutical identification numbers (PZNs) into five designated spaces. The PZN is used for almost all products sold by German pharmacies. It is printed on the drug package and precisely identifies the drugs with respect to package size, dosage, manufacturer, listed price, etc. Prices were tracked via PZNs using the official standard price list ‘Lauer-Taxe’ (as of August 2012), which provides data for all pharmaceuticals dispensed in German pharmacies [10]. In the present analysis, only those OTC drugs used were included where the prices were clearly identifiable via the PZNs.

For comparison, also the distribution of the prices of all medicinal products with non-prescription status currently listed in the ‘Lauer-Taxe’ (as of November 2016) was analyzed (*n* = 49,909 listed pharmaceuticals).

The reported drugs were subdivided into prescription drugs and OTC drugs (available without a physician’s prescription) according to the German Ordinance on Prescription-Only medicinal products [11]. Maternal and paternal education were classified into three levels (low, middle, high) based on the completed years of schooling (low = less than 10 years; middle = exactly 10 years; high = more than 10 years). The household income level was classified based on the median equivalent income (MEI) of 2012 (€1633 net/month), where the household members are weighted according to the new scale of the Organisation for Economic Co-Operation and Development [12]. The income cut-offs correspond to the defined cut-off for poverty, which is 60% of the MEI [13]. Three income levels were defined (low: ≤ 60% of MEI; middle: 60–100% of MEI; high: > 100% of MEI).

### Statistical analysis

For the statistical analysis, the software package SAS was used (SAS Institute Inc., Cary, NC, USA, version 9.3). Bivariate associations were tested with Pearson’s Chi^2^ test. Odds ratios (ORs) and their 95% confidence intervals (CIs) for the use of the defined higher priced OTC drugs were obtained from a multivariate logistic regression model. The calculated ORs show the probability of having the defined category outcome = 1 for the stratum of interest, compared with the defined category outcome = 1 in the according reference stratum. All logistic models were calculated with the SAS option “Param = ref. missing”, which considers also the missing values as a separate “stratum”.

As outcomes, two types of users of higher priced OTC drugs within the last 4 weeks were addressed:

- First, adolescents taking at least one OTC drug costing €10 or more.
- Second, adolescents taking at least one OTC drug costing €25 or more.

Gender, place of residence, maternal/paternal education level, household income status, and presence of a chronic disease were included as independent variables in the logistic regression model. Chronic disease was defined having reported one or more of the following diseases diagnosed by a physician within the preceding 5 years: hay fever, perennial allergic rhinitis, food allergy, atopic dermatitis, and asthma. Additionally, also those participants having mentioned a self-reported chronic condition such as diabetes, celiac disease, etc. were defined as chronically ill.

Further information on the methodology (e.g., inclusion criteria, definition of the drug categories, classification of socioeconomic variables, etc.) is available in detail in previous publications [14, 15].

The GINIplus and LISAplus cohorts obtained approval from the respective ethics committees (Bavarian Medical Council, University of Leipzig, Medical Council of North Rhine-Westphalia). Furthermore, written informed consent was given by the participants’ parents/legal guardians and by participants.

## Results

**Fig. 1:**
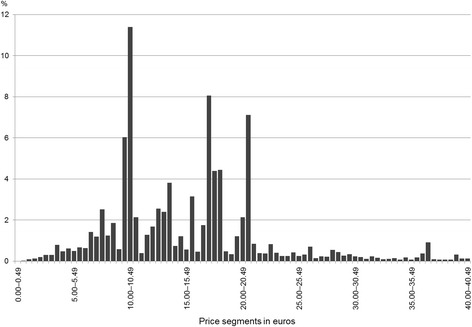
Price distribution (by price segments of 0.50 euros each) for all (*n* = 49,909) over-the-counter (OTC) drugs marketed in Germany (drugs costing up to €40.49 displayed only, representing 92% of all 49,909 drugs)

**Fig. 2:**
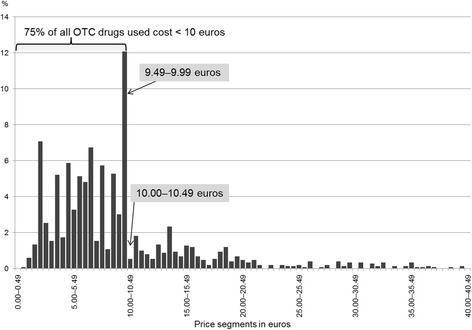
Price distribution (by price segments of 0.50 euros each) for all (*n* = 1499) over-the-counter (OTC) drugs used by adolescents of the German GINIplus and LISAplus studies (drugs costing up to €40.49 displayed only, representing 99% of all 1499 drugs used)

Questionnaires on drug use were returned from 4677 adolescents (response rate: 76.8%). Exactly 3873 drugs had been used by the study participants within the past 4 weeks. The majority (*n* = 2694) were OTC drugs and accounted for 70% of all drugs used. For 1499 OTC drugs that were eligible for the further analysis, listed prices were tracked by the reported PZN number. The mean price per OTC drug was €9.75 [95% CI: €9.27–10.22; standard deviation (SD): 9.44], ranging from €0.94 to €158.98. The distribution of the prices of all OTC drugs (*n* = 49,909) listed in the official ‘Lauer-Taxe’ is displayed in Fig. 1. 19.5% of the listed OTC drugs cost less than €10, while 15.2% cost ≥ €25. In comparison with all OTC drugs of the ‘Lauer-Taxe’, most (74.6%; *n* = 1118) of the OTC drugs used by 15-year-old adolescents had listed prices of less than €10, whereas 74 (4.9%) OTC drugs were sold at prices of €25 or more (Fig. 2). A high percentage (12.1%) of OTC drugs cost between €9.50 and €9.99, the price segment just below €10. Only eight OTC drugs (0.5%) were in the next, same-sized price segment between €10.00 and €10.49.

There was a noticeable variation in mean OTC drug prices in the different therapeutic categories. Whereas conventional drugs with chemical active ingredients (e.g. ibuprofen or paracetamol) cost €7.23 [95% CI: 6.78–7.68; SD: 6.97] on average (range: €0.94–158.98), homeopathic drugs were sold at €12.67 [95% CI: 11.41–13.94; SD: 9.55] on average (range: €3.70–116.69), and herbal drugs (range: €1.99–94.45) had a mean price of €14.99 [95% CI: 13.41–16.56; SD: 11.41].

The average price of OTC drugs (Table 1) used by male adolescents (€10.14; 95% CI: 9.27–11.01; SD: 11.39) was higher than the mean price of OTC drugs used by female adolescents (€9.44; 95% CI: 8.92–9.95; SD: 7.57). OTC drugs that were purchased by adolescents living in Munich had the highest mean prices (€10.74; 95% CI: 9.97–11.52; SD: 10.80), about €2 above the price level of OTC drugs that were bought in Wesel (€8.65; 95% CI: 7.98–9.32; SD: 8.02), where the mean drug price was lowest.

**Table 1:** Cohort structure and mean over-the-counter (OTC) drug prices with 95% confidence intervals (CI)

	n adolescents^a^	n OTC drugs used^b^	mean OTC drug price in €	95% CI
Total cohort	4677	1499	9.75	(9.27–10.22)
Gender				
Male	2365	656	10.14	(9.27–11.01)
Female	2312	843	9.44	(8.92–9.95)
Study area				
Munich	2343	752	10.74	(9.97–11.52)
Leipzig	414	122	9.19	(7.87–10.51)
Bad Honnef	188	72	8.66	(7.32–10.00)
Wesel	1732	553	8.65	(7.98–9.32)
Maternal educational level				
Low	482	113	9.35	(7.71–10.99)
Medium	1731	572	9.13	(8.34–9.92)
High	2277	746	10.20	(9.52–10.88)
Paternal educational level				
Low	926	240	8.12	(7.41–8.83)
Medium	989	294	10.13	(9.02–11.24)
High	2458	869	10.17	(9.47–10.86)
Household income				
Low	686	206	9.18	(7.92–10.44)
Medium	1526	534	9.44	(8.77–10.11)
High	1817	583	10.24	(9.34–11.14)
Chronic disease				
No	3425	1007	9.41	(8.83–10.00)
Yes	1239	479	10.40	(9.56–11.25)

^a^Owing to missing values, the strata may not add up to the total of n = 4677 participants

^b^Only those OTC drugs with clearly identifiable prices were considered for analysis

Higher maternal or paternal education was correlated with higher mean prices for OTC drugs used (e.g., lowest paternal education level (€8.12; 95% CI: 7.41–8.83; SD: 5.56) vs. highest paternal education level (€10.17; 95% CI: 9.47–10.86; SD: 10.40)).

On average, adolescents from the lowest income group bought cheaper OTC drugs (€9.18; 95% CI: 7.92–10.44; SD: 9.15) than adolescents from the highest income group (€10.24; 95% CI: 9.34–11.14; SD: 11.08). This association of higher drug prices with higher educational or income levels was also visible within specific drug categories (conventional drugs, homeopathic drugs, herbal drugs) or even within the very narrowly defined category of drugs containing ibuprofen only (the most mentioned active ingredient), where children from the highest socioeconomic level used higher priced ibuprofen containing drugs (e.g. lowest income level (€4.82; 95% CI: 4.37–5.27; SD: 1.22) vs. highest income level (€5.94; 95% CI: 5.38–6.51; SD: 2.86)).

Generally, adolescent users of OTC drugs priced at ≥ €10 were significantly more likely (tested with Chi^2^) to reside in Munich (*p* = 0.0060) and to have a father (*p* < 0.0001) with the highest education level. However, in the bivariate test, general use of OTC drugs (regardless of the price) was also positively associated (tested with Chi^2^) with residing in Munich (*p* = 0.0459), paternal education level (*p* = 0.0002), or maternal education level (*p* = 0.0003). The crude ORs for the association between the outcomes and the independent variables are shown in Table 2.

**Table 2:** Crude odds ratios for over-the-counter (OTC) drug use by price category

	Crude odds ratio of utilization (and 95% confidence interval)					
	All OTC drugs		OTC drugs ≥ €10		OTC drugs ≥ €25	
Gender						
Male	Reference		Reference		Reference	
Female	1.50***	(1.33–1.70)	1.27*	(1.00–1.61)	0.93	(0.56–1.54)
Study area						
Munich	Reference		Reference		Reference	
Leipzig	0.85	(0.68–1.06)	0.83	(0.54–1.26)	0.53	(0.19–1.50)
Bad Honnef	1.00	(0.73–1.37)	0.92	(0.51–1.64)	0.29	(0.04–2.14)
Wesel	0.83**	(0.73–0.95)	0.62**	(0.47–0.81)	0.45**	(0.24–0.82)
Maternal educational level						
Low	Reference		Reference		Reference	
Medium	1.44**	(1.14–1.82)	1.63	(0.98–2.72)	0.97	(0.39–2.43)
High	1.59**	(1.27–1.99)	1.95*	(1.19–3.20)	1.02	(0.42–2.48)
Paternal educational level						
Low	Reference		Reference		Reference	
Medium	1.10	(0.90–1.34)	1.55	(1.00–2.39)	2.84*	(1.03–7.83)
High	1.37**	(1.16–1.62)	2.20**	(1.51–3.19)	2.81*	(1.10–7.18)
Household income						
Low	Reference		Reference		Reference	
Medium	1.24*	(1.02–1.51)	1.46	(0.98–2.17)	0.85	(0.38–1.91)
High	1.27*	(1.05–1.54)	1.32	(0.89–1.95)	1.14	(0.53–2.43)
Chronic disease						
No	Reference		Reference		Reference	
Yes	1.65***	(1.44–1.89)	1.88***	(1.48–2.40)	2.00**	(1.19–3.35)

Significant at *p < 0.05 **p < 0.01 ***p < 0.0001

In the multivariate logistic regression model that contained all independent variables (Table 3), those living in Munich (OR = 0.72; Wesel vs. Munich) and those having higher educated fathers (OR = 2.00; high education level vs. low education level) were significantly more likely to use OTC drugs costing €10 or more. Both associations were even stronger, when looking at OTC drugs costing €25 or more, as the ORs for using OTC drugs costing ≥ €25 were 0.50 (Wesel vs. Munich) and 3.02 (highest paternal education level vs. lowest paternal education level), respectively.

**Table 3:** Adjusted odds ratios of over-the-counter (OTC) drug use by price category

	Adjusted odds ratio of utilization (and 95% confidence interval)					
	All OTC drugs		OTC drugs ≥ €10		OTC drugs ≥ €25	
Gender						
Male	Reference		Reference		Reference	
Female	1.52***	(1.34–1.73)	1.27*	(1.00–1.61)	0.90	(0.54–1.49)
Study area						
Munich	Reference		Reference		Reference	
Leipzig	0.87	(0.69–1.10)	0.85	(0.55–1.32)	0.49	(0.17–1.42)
Bad Honnef	1.09	(0.79–1.50)	1.00	(0.55–1.81)	0.32	(0.04–2.35)
Wesel	0.93	(0.81–1.08)	0.72*	(0.54–0.97)	0.50*	(0.26–0.96)
Maternal educational level						
Low	Reference		Reference		Reference	
Medium	1.35*	(1.06–1.72)	1.31	(0.77–2.23)	0.70	(0.27–1.81)
High	1.35*	(1.05–1.73)	1.29	(0.75–2.20)	0.56	(0.21–1.50)
Paternal educational level						
Low	Reference		Reference		Reference	
Medium	1.03	(0.83–1.27)	1.43	(0.91–2.25)	3.20*	(1.11–9.21)
High	1.23*	(1.01–1.50)	2.00**	(1.32–3.04)	3.02*	(1.07–8.53)
Household income						
Low	Reference		Reference		Reference	
Medium	1.15	(0.94–1.42)	1.21	(0.80–1.81)	0.69	(0.30–1.58)
High	1.07	(0.86–1.33)	0.85	(0.56–1.31)	0.73	(0.32–1.67)
Chronic disease						
No	Reference		Reference		Reference	
Yes	1.66***	(1.44–1.90)	1.87***	(1.46–2.38)	1.95*	(1.16–3.28)

Significant at *p < 0.05 **p < 0.01 ***p < 0.0001

Due to the outcome definition, participants that took OTC drugs costing ≥ €25 belong to the group taking OTC drugs ≥ €10 as well. A sensitivity analysis excluding those 61 users belonging to both groups showed only slight differences for the OR values between adolescents (n = 237) taking OTC drugs with prices €10–24.99 and those adolescents (n = 298) reporting use of OTC drugs costing ≥ €10.

## Discussion

The findings of this study imply that adolescents’ acceptance to use OTC drugs with prices beyond a certain price threshold might be limited, if they are not covered by statutory health insurance. In Germany, for people aged ≥12 years, only few OTC drugs for very specific indications are included in the list of reimbursable OTC drugs. Currently, this list of exemptions presented by the Federal Joint Committee mentions about 40 active ingredients or herbal extracts only [16]. Most (75%) of the OTC drugs used by the adolescents of the present study cost less than €10, in contrast to the price distribution of all OTC drugs available on the German market, where only about 20% of the OTC drugs have listed prices of less than €10. Additionally, many (12.1%) of the OTC drugs used were from the 50-cent price segment just below €10, while only 0.5% were from the following same-sized price segment just above €10. This drop at the €10-threshold was not visible in the distribution for all OTC drugs marketed in Germany. It might therefore be concluded that the relatively moderate price of €10 for an OTC drug could generally represent a psychological barrier that may discourage consumers from buying higher priced non-reimbursable OTC drugs.

The results of the present analysis also indicate that adolescents’ use of higher priced OTC drugs may be associated with socioeconomic background such as place of residence or paternal educational level. Interestingly, mothers’ educational level was associated with general OTC drug use only, but no association was found with the use of higher priced OTC drugs. This may be explained such that mothers, compared with fathers, may care less about the price of self- medication, once mothers have decided that their children would need an OTC drug. There may also be an association with income, but further research is needed to confirm this on a significant basis, as in this study the mean price of OTC drugs was higher in the higher income levels, but the Chi^2^ test or the logistic regression model did not reveal significant associations at *p* < 0.05 for the use of higher priced OTC drugs (costing ≥ €10 or ≥ €25, respectively) with household income. In a sensitivity analysis, where the place of residence was excluded from the logistic regression model, household income was also not associated with the use of higher priced OTC drugs. However, those living in Munich, a rather wealthy region in Germany, were significantly more likely (compared with Wesel) to use an OTC drug costing ≥ €10 or ≥ €25. Also, having a father from the highest education level (compared with the lowest education level) increased significantly the probability to use an OTC drug costing ≥ €10 (OR = 2.00) or ≥ €25 (OR = 3.02).

Nevertheless, prices for OTC drugs that have to be paid for out of pocket are not an issue as long as cheaper alternatives of comparable quality are available or OTC drugs are covered by health insurance. On the other hand, it should also be considered that higher priced but medically advisable OTC drugs are possibly not used if they have to be paid for out of pocket, especially in view of adolescents from the lowest socioeconomic level. The ‘€10-barrier’ could have an impact on the use of e.g. non-reimbursable herbal drugs (mean price: €14.99), which may be less attractive just because they cost more than €10 on average. But also with regard to other drug categories (e.g. emergency contraceptives, recently switched to OTC status and costing roughly €18 (levonorgestrel) or €30 (ulipristal acetate) in Germany), the price should not be the predominant factor that determines whether a drug is used or not. It should also be pointed out that there is a difference between an OTC drug’s price and e.g. expenditures for OTC products within a defined period. In the situation of deciding about buying an OTC drug or not, the potential purchaser presumably does not have in mind, how much he already spent for his medication within a defined preceding time period. If economic aspects should play a roll, it is likely, that the purchaser’s (spontaneous) decision to buy or not to buy an OTC drug will depend on the drug’s (high) price rather than on the fact that his e.g. monthly “personal budget” for expenditures on OTC drugs would still allow or no longer allow him to buy this specific OTC drug. However, the number of OTC drug packages used might also be linked with adolescents’ purchase behaviour, as it may be hypothesized that adolescents who already bought two, three, or more OTC drug packages could have been more reluctant to spend money for further OTC drugs within the observation period of 4 weeks. However, in this study, the mean number of OTC drug packages used was highest among those adolescents who took at least one OTC drug costing ≥€25 (mean = 3.03 OTC drugs), compared with 2.47 OTC drug packages in the mean for those using OTC drugs costing ≥€10, and 1.81 OTC drug packages on average for all OTC drug users (no price threshold). This may further support the hypothesis that use of higher priced OTC drugs is linked with higher socioeconomic background, as e.g. those adolescents with higher educated fathers not only are more likely to use higher priced OTC drugs, but simultaneously also use more OTC drug packages on average.

Of course, these results may also have implications on prescription-only drugs that are not covered by social security. For instance, a study conducted in the United States [17] found that, on average, women using oral contraceptives spent $15 per cycle on average, but 50% of those women spent not more than $10 per cycle (possibly, women would be willing to spend about $5 more just for the convenience to get oral contraceptives over-the-counter instead of having it prescribed by a physician). It seems to be evident that there is a price limit (which, however, may vary by country, socioeconomic level, therapeutic category, etc.) also for medically relevant drugs, if they have to be paid out of pocket.

OTC drugs should not be considered as second class medicinal products compared to prescription-only drugs. It might be meaningful to screen the available OTC drugs and to define, which of them are medically relevant, for instance drugs for fungal skin or nail infections, remedies against lice infestation, emergency contraceptives (in theory, reimbursable up to the age of 20 years, if prescribed by a physician), antihistamines such as cetirizine or loratadine, etc. Those relevant OTC drugs might then either be reimbursed for all age groups, or on the other hand, it should somehow be ensured that the drugs’ prices do not affect accessibility to those remedies. In the meantime, this issue is also being discussed on a political level [18]. However, it remains elusive, why medically relevant OTC drugs should be covered by statutory health insurance up to the age of 12 years only.

This study has strengths and limitations. To our knowledge, this is the first study analyzing directly the association of prices of (presumably) self-medicated OTC drugs with the users’ socioeconomic background. The analysis is based on recent data from two large population-based German birth cohorts that, however, cannot be considered to be representative for Germany as a whole.

A limiting factor of the above-mentioned results is that prices for all OTC drugs are freely calculable in Germany and only the listed prices were considered, which may vary from the actual prices paid in the pharmacies. The distribution of the prices of all marketed OTC drugs in Germany was available for 2016 only. For direct comparison with the users’ price distribution, prices for all OTC drugs available in Germany based on the 2012 ‘Lauer-Taxe’ would have been preferable, as also the tracked prices in this analysis of those OTC drugs actually used were from 2012. Nevertheless, the increase of the OTC drugs’ prices since 2012 may be estimated at about 1.5% per year only [19] and the impact of inflation on the price distribution may therefore be very limited. No data were available on how many of the included OTC drugs were actually paid out of pocket, had been prescribed on a reimbursable prescription form by physician, or had been reimbursed after the purchase over the counter (some statutory insurance companies reimburse the costs for specific OTC drugs when presenting the receipt), as this information was not assessed with the questionnaire. Furthermore, the proportion of privately insured adolescents in this study is not known (private health insurance companies may cover more OTC drugs compared with statutory health insurance agencies). However, privately insured individuals in Germany only account for about 13% [20] of all insured people, but this proportion may be slightly higher in the present study, due to the overrepresentation of the socioeconomically higher strata compared with the German mean. Data collection for this study stretched over 46 months and was distributed almost evenly over the four seasons (with slight peaks in April and June). Sensitivity analysis showed that the use of higher or lower priced drugs was not influenced by seasonal aspects relevantly. A sensitivity analysis was also performed for the presence of chronic disease. No relevant difference (compared with the full models) was visible, if the independent variable for “chronic disease” was removed from the logistic regression models. Also, the inclusion of missing values into the logistic regression models with the SAS option “Param = ref. missing” may have had an impact on the magnitude of the ORs. However, compared to the SAS output without using this option, the difference between the respective ORs was small. Finally, the cohort composition with respect to gender, parental education, household income, and presence of chronic disease differed between Munich, Leipzig, Bad Honnef, and Wesel. Hence, the magnitude of the independent variables’ predicting effect may vary between the four included study areas. However, the difference between e.g. Munich and Wesel, the study areas 87% of the adolescents came from, was limited.

## Conclusions

The majority (75%) of OTC drugs used by adolescents cost less than €10. Based on the results of this study, it may be finally concluded that higher prices of non-reimbursable OTC drugs may discourage potential purchasers from buying also medically relevant OTC drugs. Especially those from a weaker socioeconomic background may use less medically advisable OTC drugs, if they have to be paid for out of pocket. Therefore, the list of non-reimbursable OTC drugs in Germany should be reassessed and screened for medically relevant OTC drugs, which might then be covered by statutory health insurance companies again.

## Abbreviations

CI: Confidence interval
MEI: Median equivalent income
OR: Odds ratio
OTC: Over-the-counter
PZN: Pharmaceutical identification number
SD: Standard deviation
